# Does implementation of the PECARN rules for minor head trauma improve patient-centered outcomes in a lower resource emergency department: a retrospective cohort study

**DOI:** 10.1186/s12887-020-02328-x

**Published:** 2020-09-17

**Authors:** Rasha D. Sawaya, Cynthia Wakil, Adonis Wazir, Sami Shayya, Iskandar Berbari, Rawan Safa, Maha Makki, Mahdi Hamade, Hani Tamim

**Affiliations:** 1 Department of Emergency Medicine, American University of Beirut Medical Center, Beirut, Lebanon; 2 Faculty of Medicine, American University of Beirut, Beirut, Lebanon; 3 Faculty of Medicine, Clinical Research Institute, American University of Beirut, Beirut, Lebanon; 4 Department of Internal Medicine, American University of Beirut, Beirut, Lebanon

**Keywords:** Minor head trauma, Clinically important traumatic brain injury, PECARN prediction rules, CT imaging, Pediatric patients

## Abstract

**Background:**

Managing children with minor head trauma remains challenging for physicians who evaluate for the need for computed tomography (CT) imaging for clinically important traumatic brain injury (ciTBI) identification. The Pediatric Emergency Care Applied Research Network (PECARN) prediction rules were adopted in our pediatric emergency department (PED) in December 2013 to identify children at low risk for ciTBI. This study aimed to evaluate this implementation’s impact on CT rates and clinical outcomes.

**Methods:**

Retrospective cohort study on pediatric patients with head trauma presenting to the PED of the American University of Beirut Medical Center in Lebanon. Participants were divided into pre- (December 2012 to December 2013) and post-PECARN (January 2014 to December 2016) groups. Patients were further divided into < 2 and ≥ 2 years and stratified into groups of low, intermediate and high risk for ciTBI. Bivariate analysis was conducted to determine differences between both groups.

**Results:**

We included 1362 children of which 425 (31.2%) presented pre- and 937 (68.8%) presented post-PECARN rules implementation with 1090 (80.0%) of low, 214 (15.7%) of intermediate and 58 (4.3%) of high risk for ciTBI. CTs were ordered on 92 (21.6%) pre- versus 174 (18.6%) patients post-PECARN (*p* = 0.18). Among patients < 2 years, CT rates significantly decreased from 25.2% (34/135) to 16.5% (51/309) post-PECARN (*p* = 0.03), and dropped in all risk groups but only significantly for low risk patients from 20.7% (24/116) to 11.4% (30/264) (*p* = 0.02). There was no significant decrease in CT rates in patients ≥2 years (20% pre (58/290) vs 19.6% post (123/628), *p* = 0.88). There was no increase in bounce back numbers, nor in admission rates or positive CT findings among bounce backs.

**Conclusions:**

PECARN rules implementation did not significantly change the overall CT scan rate but reduced the CT scan rate in patients aged < 2 years at low risk of ciTBI. The implementation did not increase the number of missed ciTBI.

## Background

Head trauma commonly occurs in childhood and accounts for a large percentage of Pediatric Emergency Department (PED) visits worldwide [1, 2]. In the United States (US), traumatic brain injury (TBI) results in more than 50,000 deaths and over 200,000 hospital admissions every year [1], about 125,000 of which end up with disability [3]. Among pediatric patients, however, most head trauma cases are minor and only few require further interventions [4, 5]. Nevertheless, given the acute [6] and long-term [7, 8] sequelae associated with TBI, rapid identification of children who may require acute interventions for clinically-important traumatic brain injury (ciTBI) is of crucial importance in the PED. The group which poses the most clinical equipose and that has been extensively studied are the children who present with minor TBI. We define minor TBI as patients with head trauma who have a GCS ≥14.

Computed Tomography (CT) imaging is highly sensitive for the identification of ciTBI and remains the gold standard diagnostic tool for the evaluation of head trauma patients [9]. However, the use of diagnostic radiation has been associated with an increased risk of cancer in children, whose tissues are more susceptible to radiation-related cancer than adults [10, 11]. Moreover, the overuse [12–14] and variability [15, 16] in CT imaging of children with head trauma between clinicians and hospitals appear to be unrelated to the frequency of positive CT scans and ciTBI [4, 16–19]. As such, for the management of these children, PED physicians should better evaluate the need for CT imaging for ciTBI identification in order to limit radiation exposure and optimize resource utilization [20].

In pediatric patients with head trauma, particularly young children, individual ciTBI predictors lack specificity, which makes it difficult for PED physicians to identify and predict the severity of TBI. The Pediatric Emergency Care Applied Research Network (PECARN) developed clinical prediction rules for ciTBI in pediatric patients with minor head trauma, hereafter referred to as the PECARN rules, which were derived and validated to identify children at very low risk of ciTBI [4]. They include 6 different predictors for pediatric patients that are either less than or more than or equal to 2 years of age. Upon several validations in various settings, these rules were shown to have 100% negative predictive value for ciTBI with a sensitivity of 100% [4, 5, 21, 22]. Because of their good discrimination for ciTBI among children with head trauma, they were adopted in many EDs in an attempt to reduce head CT rates without affecting patient outcomes [23–25]. As shown by Dayan et al. in their large prospective multicenter study, the implementation of the PECARN rules results in safe, but variable, decreases in the use of CT, depending on the setting and method of implementation [23–25]. In some EDs, however, despite successful implementation with high adherence and medical staff satisfaction, CT scan rates remained unchanged [20, 26]. In settings with highly accurate clinician judgment, implementation of the PECARN rules may have limited impact on improving detection of ciTBI [27].

In our institution, a Middle Eastern tertiary care academic center, the attending physicians who typically evaluate cases of pediatric trauma have a variety of training backgrounds, which is the case in most EDs in Lebanon [28]. All pediatric patients with minor trauma are generally seen in our ED’s “low-acuity” section along with minor adult cases, where 24-h attending physicians are mostly specialized in Family Medicine or Surgery and only occasionally in Emergency Medicine. In order to standardize the care provided by the different specialists, we implemented the PECARN rules in December 2013. As opposed to previous studies on the impact of the utilization of the PECARN rules [5, 20–25, 27, 29], the implementation of these in our study took place in a setting that lacked Quality Improvement (QI) and administrative support, which we considered for the purpose of this study as a limited resource setting.

This study aimed to evaluate the impact of this implementation on the medical care provided in our PED despite the resource constraints, by measuring the changes in head CT scan rates before and after implementation as well as PED length of stays, missed ciTBIs and patient bounce backs.

## Methods

### Study design and setting

This was a retrospective cohort study conducted on pediatric patients presenting with minor head trauma to the ED of the American University of Beirut Medical Center (AUBMC), a large tertiary care center in Lebanon. Ethical approval was obtained from the Institutional Review Board at AUBMC under the protocol number [BIO-2017-0452]. The selection of the study period was imposed by the implementation of the PECARN rules in December 2013. The study period was thus composed of one year before implementation, from December 1st 2012 to December 31st 2013, to include all seasons, and 3 years after implementation, from January 1st 2014 to December 30th 2016, to monitor the change in CT imaging rates over time. During this study’s period, all patient documents in the ED at the AUBMC were on paper and not electronic. Documents were scanned and could only be reviewed on the Electronic Health Records (EHR) of the AUBMC. Typically, in the ED, an order was written and signed by a physician after having examined and assessed the patient; this order would be executed by nurses. When a CT imaging order was placed, nurses would call the radiology department to inform them of the CT order. The patient is then transported to the radiology department to undergo CT imaging and results are reported by the attending radiologist.

### Study population

The eligible participants for this study were identified by the decision support unit, which is part of the Electronic Health Records (EHR) at AUBMC. In order to identify the largest number of patients and minimize selection bias, we screened all patients, 0 to 18 years of age, who presented to the ED between the 1st of December 2012 and 30th of December 2016, and we reviewed the charts of all patients with the following characteristics to screen them for inclusion:

- Any patient with an ED discharge diagnosis or hospital admission diagnosis of any head related injury (Minor head trauma, head trauma, concussion, head injury, traumatic brain injury, head bleed, head laceration, intracranial hemorrhage, subdural hematoma, head hematoma, cerebral contusion, head contusion, brain contusion, skull fracture), any head injury related complication (loss of consciousness, decreased level of consciousness, comatose, intubation), any mechanism of injury that raises suspicion of head injury (fall, slip, bump, motor vehicle accident, trauma, collision, assault, hit, fight, sports injury, pedestrian struck), and other complaints that may involve concomitant head trauma (loss of consciousness, abuse, any bone fracture, nasal bleeding, bleeding, hemorrhage, broken teeth, eye ecchymosis, head laceration, any neck injury).
- Any patient who had a head CT, orbital CT, facial CT or skull X-rays done in the ED.
- Any patient who was seen in the ED and required any Intensive Care Unit (ICU) (Pediatric ICU, Surgical ICU, NeuroSurgical ICU, NeonatalI CU) admission.

Subsequently, we included all patients aged 0 to 18 years presenting to the PED with head trauma between the 1st of December 2012 and 30th of December 2016. We excluded patients with trivial injury mechanisms, which included ground-level falls or running into stationary objects, and those with no signs or symptoms of head trauma other than lacerations or abrasions. We also excluded patients with penetrating trauma, a Glasgow Coma Scale (GCS) score < 14, neurologic or bleeding disorders, known brain tumors, ventricular shunts, and those presenting after evaluation and imaging for head trauma at another hospital [20]. As our ED adopted the evidence-based PECARN clinical prediction rules for minor head trauma in December 2013, participants were divided into pre- (1st December 2012 to 31st December 2013) and post-PECARN (1st January 2014 to 30th December 2016) groups. Based on the risk stratification algorithm from the PECARN study [4], the study population was stratified into three groups at risk for ciTBI according to the PECARN clinical prediction rules (i.e. very low, intermediate, and high-risk). For both age groups, the rules included severe injury mechanism plus 5 additional clinical predictors. For children younger than 2 years, the following clinical predictors were included in the rule in addition to severe injury mechanism: altered mental status, non-frontal scalp hematoma, loss of consciousness for 5 s or greater, palpable skull fracture, not acting normally per parents. For children 2 years or older, the following clinical predictors were included in addition to severe injury mechanism: altered mental status, any loss of consciousness, history of vomiting, clinical signs of basilar skull fracture, severe headache. ciTBI was defined by any of the following descriptions [4]: death from TBI, neurosurgical intervention for TBI (intracranial pressure monitoring, elevation of depressed skull fracture, ventriculostomy, hematoma evacuation, lobectomy, tissue debridement, dura repair, or other interventions), intubation for more than 24 h for TBI, hospital admission for ≥2 nights for TBI with TBI findings on CT imaging, hospital admission for TBI corresponded to admission for persistent neurological symptoms or signs such as persistent alteration in mental status, recurrent emesis due to head injury, persistent severe headache, or ongoing seizure management.

### Implementation of PECARN rules

At implementation, in December 2013, all attending physicians in the PED were educated about the PECARN rules, through a PowerPoint presentation that was offered during the general ED department meeting at AUBMC. Residents and rotating trainees were also educated about the rules every year, via lectures. Posters of the PECARN rules were also placed in the ED for reference. As opposed to previous studies [5, 20–23, 25, 27, 29, 30], the implementation of the PECARN rules in our study took place in a limited resource setting that lacked administrative and educational resources to assist in QI initiatives. This implementation was conducted with no Pediatric Emergency Medicine trained physicians, no QI team in place and less experience in specialized QI intervention efforts.

### Data collection

The data collection team was composed of research assistants (RA) and medical graduates, all with CITI certification and familiar with our medical charts, who were not blinded to the study hypothesis. The principal investigator (PI) and lead RA developed a data collection manual corresponding to the data collection sheet for this study. This manual had the definitions of all the required variables (including all their potential corresponding terminology in the charts) and their corresponding locations on the EHR of the study participants. The data collection team then performed a pilot data collection on a small number of charts and discussed thereafter any concerns regarding the data collection process to evaluate the need to modify the data collection manual accordingly. After training, the data collection team proceeded with the data collection process. The team met regularly thereafter to discuss any potential questions or doubts they may have; consensus was reached with the PI. Finally, multiple quality checks of 15% of the charts were performed in parallel by a second reviewer, to assess the quality of the data. Collected variables included patient demographics, mechanisms of injury, symptoms and physical exam findings, as well as management and clinical outcomes. TBI on CT scan was defined by any of the following descriptions [4]: Intracranial hemorrhage or contusion, cerebral edema, traumatic infarction, diffuse axonal injury, shearing injury, sigmoid sinus thrombosis, midline shift of intracranial contents or signs of brain herniation, diastasis of the skull, pneumocephalus, skull fracture depressed by at least the width of the table of the skull.

The primary outcome of this study consisted of the rates of head CT ordered pre- and post-implementation of the PECARN prediction rules. Secondary outcomes consisted of balancing measures such as PED length of stays, neurology and neurosurgery consults, admission rates, rates of missed ciTBIs and 2-week bounce backs for symptoms and/or signs potentially related to minor head trauma.

### Data analysis

Patients in both groups were compared, and their baseline characteristics described and presented as mean ± SD for continuous variables and frequency (%) for categorical variables. On bivariate analysis, Student’s t-test was used for continuous data while Chi-square and Fisher’s exact tests were used for categorical data. All statistical analyses were performed using SPSS 24 (Statistical Package for Social Sciences). Statistical significance was set at a bilateral *p*-value of 0.05.

## Results

### Patient characteristics

**Fig. 1:**
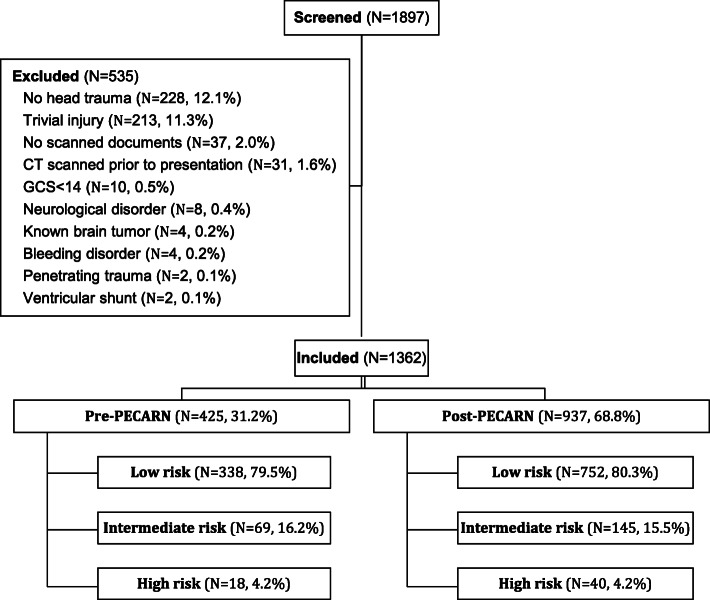
Study flowchart of pediatric patients presenting to the PED with minor head trauma

A total of 1897 pediatric patients presenting with head trauma were initally screened and 535 patients were excluded for the criteria listed in Fig. 1. Our study included 1362 patients, 425 (31.2%) of which presented pre- and 937 (68.8%) presented post-PECARN rules implementation. Our study population consisted of 1090 (80.0%) patients of low risk, 214 (15.7%) of intermediate risk and 58 (4.3%) of high risk for ciTBI, with no significant difference in risk between pre- and post- PECARN groups (*p* = 0.94) (Fig. 1). More than two thirds of our population was ≥2 years of age with no significant difference between pre- (68.2%) and post-PECARN (67.0%) groups (*p* = 0.66). In general, there were no significant differences in patient characteristics and injury presentations between pre- and post-PECARN groups except for slightly less vertigo (0.5% vs 0.0%, *p* = 0.04) and altered level of consciousness (8.0 vs 4.2%, *p* = 0.004) in the post-PECARN group (Table 1).

**Table 1:** Characteristics of patients presenting with head trauma pre- and post-PECARN rules implementation

Variables	Pre-PECARN (*N* = 425)	Post-PECARN (*N* = 937)	*p*-value
Age, in years, mean (±SD)	4.75 (± 4.67)	4.42 (± 4.44)	0.21
Age, in years, n (%)			
< 2	135 (31.8)	309 (33.0)	0.66
≥ 2	290 (68.2)	628 (67.0)	
Male, mean (±SD)	254 (59.8)	534 (57.0)	0.34
Severe mechanism of injury^a^, n (%)	16 (3.8)	35 (3.7)	0.98
Symptoms, n (%)			
Dizziness	26 (6.1)	54 (5.8)	0.8
Vertigo	2 (0.5)	0 (0.0)	0.04
Amnesia	11 (2.6)	18 (1.9)	0.43
Nausea	15 (3.5)	27 (2.9)	0.52
Vomiting	68 (16.0)	136 (14.5)	0.48
Seizure	4 (0.9)	8 (0.9)	0.87
Vision changes	3 (0.7)	13 (1.4)	0.3
Altered mental status^b^	34 (8.0)	39 (4.2)	0.004
Severe headache	9 (2.1)	19 (2.0)	0.91
LOC	20 (4.7)	39 (4.2)	0.65
LOC > 5 s	17 (4.0)	20 (2.1)	0.05
Physical Exam findings, n (%)			
Scalp Occipital/Parietal/Temporal Hematoma	19 (4.5)	44 (4.7)	0.86
Palpable skull fracture	0 (0.0)	1 (0.1)	0.5
Signs of basilar skull fracture^c^	1 (0.2)	1 (0.1)	0.57
Not acting normally as per parent	9 (2.1)	36 (3.8)	0.1
Altered mental status	17 (4.0)	38 (4.1)	0.96
GCS 14	2 (0.5)	2 (0.2)	0.7
GCS 15	417 (98.1)	920 (98.2)	
Risk Stratification, n (%)			
Low Risk	338 (79.5)	752 (80.3)	0.94
< 2	116 (34.3)	264 (35.1)	0.8
≥ 2	222 (65.7)	488 (64.9)	
Intermediate Risk	69 (16.2)	145 (15.5)	0.94
< 2	13 (18.8)	29 (20.0)	0.84
≥ 2	56 (81.2)	116 (80.0)	
High Risk	18 (4.2)	40 (4.2)	0.94
< 2	6 (33.3)	16 (40.0)	0.63
≥ 2	12 (66.7)	24 (60.0)	

^a^Severe mechanism of injury: motor vehicle crash with patient ejection, death of another passenger, or rollover; pedestrian or bicyclist without helmet struck by a motorized vehicle; falls of more than 0.9 m (if < 2 years of age) or more than 1.5 m (if more than 2 years of age); or head struck by a high-impact object

^b^Altered mental status: agitation, somnolence, repetitive questioning, or slow response to verbal communication [4]

^c^Signs of basilar skull fracture included the battle’s sign, racoon eyes, hemotympanum, cerebral spinal fluid otorrhea, or cerebral spinal fluid rhinorrhea [4]

### Management and clinical outcomes

Only 8 (1.9%) patients in the pre-PECARN group and 7 (0.7%) in the post-PECARN group were diagnosed with ciTBI (*p* = 0.09). Among patients that had CT imaging, only 8 (8.7%) pre- and 13 (7.5%) post-PECARN had positive findings on CT (*p* = 0.73). Nevertheless, significantly more written discharge instructions specifically related to head trauma were given post-PECARN rules implementation (51.0% vs 44.0%, *p* = 0.02). There was no significant difference in the number of neurology and neurosurgery consults (6.6% vs 4.4%, *p* = 0.09), ED length of stay (75.2 ± 76.6 vs 69.3 ± 68.1 min, *p* = 0.18), nor in patient disposition (96.8% vs 92.4% discharged home, *p* = 0.66) between pre- and post-PECARN groups (Table 2).

**Table 2:** Management and clinical outcomes of patients presenting with head trauma pre- and post-PECARN rules implementation

Variables, n (%)	Pre-PECARN (N = 425)	Post-PECARN (N = 937)	*p*-value
Length of stay, mean in minutes (±SD)	75.2 (± 76.6)	69.3 (± 68.1)	0.18
Diagnosed with ciTBI^a^	8 (1.9)	7 (0.7)	0.06
Neurosurgical intervention	1 (12.5)	0 (0.0)	0.33
Admission > 2 nights	4 (50.0)	5 (71.4)	0.4
Admission for persistent neurologic symptoms and signs	4 (50.0)	3 (42.9)	0.78
Consult Neurology/Neurosurgery	28 (6.6)	41 (4.4)	0.09
CT imaging	92 (21.6)	174 (18.6)	0.18
No acute post traumatic change	84 (91.3)	161 (92.5)	0.73
Positive findings^c^	8 (8.7)	13 (7.5)	0.73
Disposition			
Home	402 (94.6)	885 (94.5)	0.2
Inpatient/PICU	10 (2.4)	12 (1.3)	
Transfer/AMA	13 (3.1)	40 (4.3)	
Discharge instructions^b^	187 (44.0)	478 (51.0)	0.02
Bounce backs	31 (7.3)	65 (6.9)	0.81
CT imaging	2 (6.5)	8 (12.3)	0.38
Positive findings^c^	1 (3.2)	1 (1.5)	0.59
Disposition			
Home	30 (96.8)	61 (92.4)	0.66
Inpatient	1 (3.2)	4 (6.1)	
AMA	0 (0.0)	1 (1.5)	

^a^ciTBI: Clinically important traumatic brain injury: death, neurosurgical intervention, intubation for > 24 h, hospital admission for ≥2 nights

^b^Discharge instructions consisted of written discharge instructions related to head trauma

^c^Positive findings: intracranial hemorrhage/contusion, cerebral edema, traumatic infarction, diffuse axonal/shearing injury, sigmoid sinus thrombosis, midline shift, skull diastasis, pneumocephalus, or depressed skull fracture

### Primary outcome – CT scanning rates

CT scans were ordered on 92 (21.6%) patients pre- versus 174 (18.6%) patients post-PECARN rules implementation (p = 0.18) (Table 2).

**Fig. 2:**
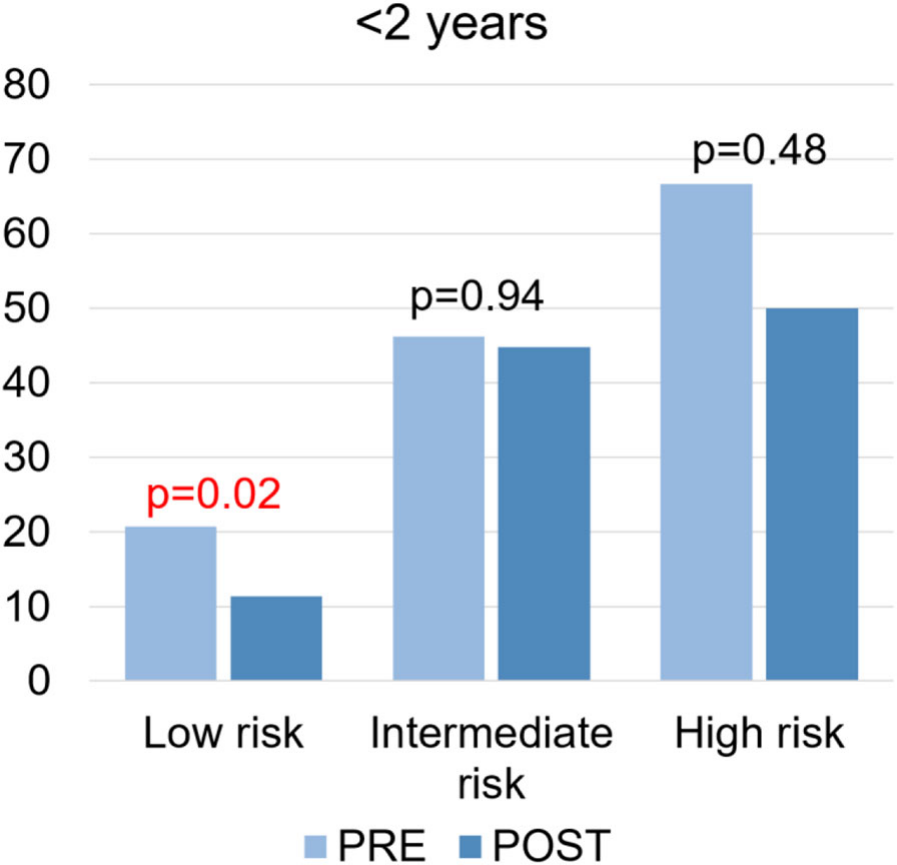
CT rates (%) in children < 2 years pre- and post-PECARN stratified by risk for ciTBI

Among patients < 2 years of age, there was a significant decrease in CT scan rates from 25.2% (34/135) pre-PECARN to 16.5% (51/309) post-PECARN (*p* = 0.03). When stratified by risk, CT scanning rates were found to drop in all risk groups, from 66.7% (4/6) to 50% (8/16) for high risk patients (*p* = 0.48), and from 46.2% (6/13) to 44.8% (13/29) for intermediate risk patients (*p* = 0.94), and only significantly for low risk patients from 20.7% (24/116) to 11.4% (30/264) (*p* = 0.02) (Fig. 2).

**Fig. 3:**
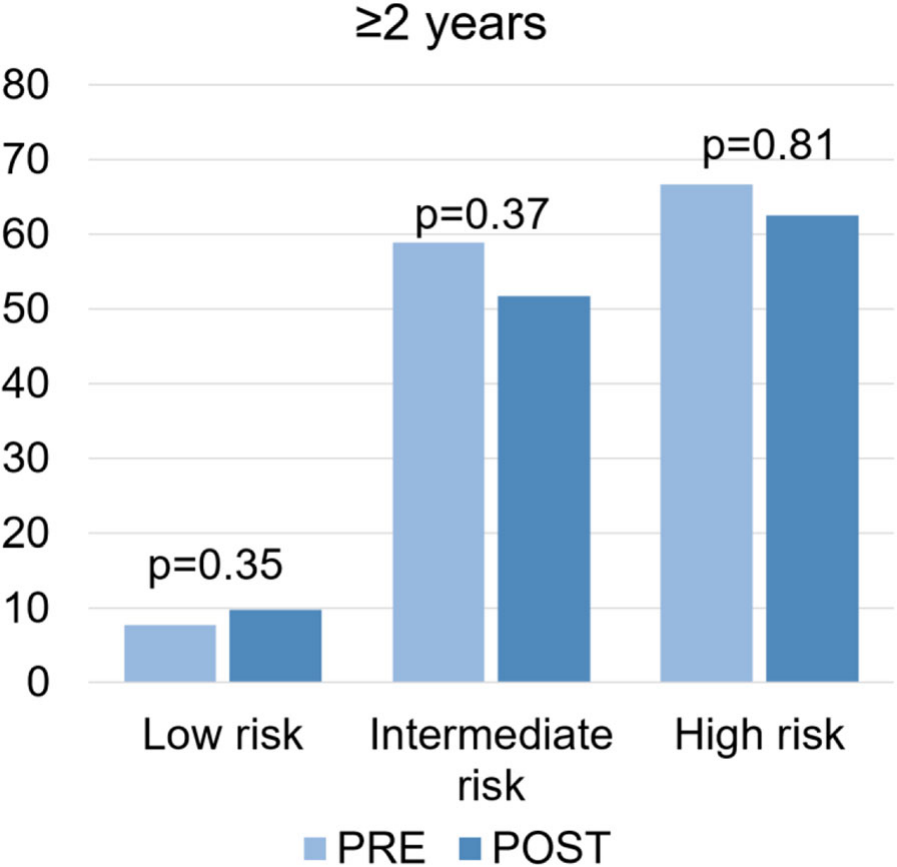
CT rates (%) in children ≥2 years pre- and post-PECARN stratified by risk for ciTBI

Among patients ≥2 years, there was no significant decrease in CT scan rates between pre and post groups (20% pre (58/290) vs 19.6% post (123/628), *p* = 0.88). When stratified by risk, a slight increase in CT scanning rates was observed among low risk patients (7.7% pre (17/222) vs 9.8% post (48/488), *p* = 0.35) and a decrease was observed in intermediate (58.9% pre (33/56) vs 51.7% post (60/116), *p* = 0.37) and high risk patients (66.7% pre (8/12) vs 62.5% post (15/24), *p* = 0.81) (Fig. 3).

### Secondary outcomes

There was no observed increase in the number of bounce backs between pre- and post-PECARN groups (7.3% vs 6.9%, p = 0.81). Among those who bounced back, CT scans were ordered on 6.5% of patients pre-PECARN and 12.3% of patients post-PECARN (*p* = 0.38); and no significant change in positive CT findings and patient disposition was noted (Table 2). Of the bounce backs, 5 children were subsequently admitted to the hospital for observation. One had a subdural hematoma, 2 had concussions and 2 presented with vomiting that was later diagnosed as acute gastroenteritis. None of them had any further complications, required surgery or died.

## Discussion

In the Middle East, the epidemiology of ciTBI and CT imaging rates of children presenting to the PED with head trauma remains understudied [31, 32]. This study evaluating the impact of PECARN rules implementation in the PED of the AUBMC in Lebanon provides a better understanding of the characteristics, clinical management and outcomes of pediatric patients presenting to our institution for minor head trauma. Moreover, this is the first study to evaluate the implementation of the PECARN rules in the region and specifically in a middle-income country, without administrative resources for QI work. The main findings of a significant decrease in CT scanning rates among low risk patients less than two years of age without any adverse effect on patient outcomes, suggest that the PECARN rules reliably identify patients at low risk for ciTBI and that their implementation can safely reduce the burden of CT imaging on children with head trauma, even in settings with limited administrative and educational resources and limited implementation efforts and QI initiatives, in order to translate knowledge, implement guidelines and change practice.

In this study, only 8 (1.9%) patients before and 7 (0.7%) patients after implementation were diagnosed with ciTBI. These low incidence rates imply that the majority of head trauma cases encountered in our PED are minor and do not require any imaging. Our results are similar to those of a large US prospective study conducted by Nigrovic et al. where only 0.9% of 42,412 patients with minor blunt head trauma had a ciTBI [30]. They are also comparable to those of a French prospective study by Lorton et al. where only 0.6% of 1499 patients with minor head trauma had a ciTBI [5]. These low rates of ciTBI thus illustrate the worldwide and, more specifically, the Lebanese population’s heightened awareness and concern for ciTBI and its consequences. Patients thus tend to seek evaluation in the ED even after a minor head trauma. As such, it is essential for ED physicians to optimize their approach to this common presentation for which only a minority are at risk of a bad outcome, given the potential harm associated with CT imaging of children with head trauma [10, 11].

In our study, the baseline CT scanning rate for all included patients pre-PECARN i.e. before any intervention was of 21.6%. Interestingly, in the literature, the several studies investigating the impact of PECARN rules on CT scanning rates of pediatric patients with head trauma display different baseline rates of head CT scanning. Whereas our clinical setting is characterized by a lower overall baseline CT scan rate (21.6% vs. 35.3% in the PECARN study) when compared to the US [4, 30, 33], our CT scanning rates seem to be higher than those reported in Europe (5.1 to 8.4%) [5, 20]. Compared to the large prospective US study validating the PECARN prediction rules, our study included a higher percentage of children younger than 2 years of age and a lower proportion of cases with severe mechanisms of injury or with high-risk predictor findings for ciTBI, such as signs of altered mental status or of basilar skull fractures [4]. Moreover, the majority (80.0%) of the patients included in this study were at low risk of a ciTBI and only 4.3% of them had a high risk for ciTBI, compared to 56 and 14% of the patients enrolled in the large prospective US study, respectively [4]. Actually, in a previous Lebanese study, Habre observed that severe cases of TBI rarely reached hospitals and are thus underestimated in Lebanon [31]. These differences reflect the overall lower severity of trauma cases presenting to our PED and further emphasize the need for selective CT imaging of Lebanese children with minor head trauma. Moreover, the observed variability in baseline rates in different populations highlights the importance of this study in Lebanon as it provides a real-world understanding of how PECARN rules perform differently in different settings.

In our institution, the implementation of the PECARN rules led to a 3% decrease in CT scanning rates of children with head trauma, down to 18.6%. Despite our study’s decrease in the amount of head CTs performed on patients after PECARN rules implementation, it is quite surprising that no significant increase was seen in the frequency of positive CT findings. Among patients who were scanned, only 8.7% (pre) and 7.5% (post) had positive findings on CT. These rates of abnormal CTs are lower than previously reported rates [23], which shows that a high number of unnecessary CT scans are still being performed in our institution. In the literature, implementation studies conducted in different settings achieved mixed results with regards to changing practice. Some studies report no change between implementation and control groups [20, 26, 27], while others report consistent and substantial decreases in CT imaging rates [23–25]. The change in CT scanning rates appears to be influenced by the baseline CT rates [16, 19, 34], the preexisting clinician accuracy [27], the medico-legal climate, the inclination for shared decision making with families [25] and the availability of observation units for conservative watchful waiting on intermediate risk patients [20]. As such, in settings such as the US and Canada with high baseline CT rates and variability between CT rates [16, 19], clinical decision rules may contribute to a safe reduction in CT rates [24, 25] but perhaps not in other settings with low CT rates or high clinician accuracy as has been shown in Italy [20] or Australia [29, 34, 35] and in our study.

Nevertheless, although our baseline rates are comparable to those of a recent QI study conducted in the US, the implementation of PECARN guidelines in our PED had less of an impact on CT use when compared to results reported by Nigrovic et al. consisting of a CT scan decrease from 21 to 15% after implementation and down to 9% through individual provider feedback [24]. Most published studies showing a positive impact from the implementation of the PECARN rules were conducted in developed countries [5, 20–22], with adequate administrative resources, or conducted specifically as QI projects [23, 30]. According to previous studies, a CT rate of less than 15% is achievable for all children with minor blunt head trauma [20, 24, 25]. Specifically, Nigrovic et al. significantly decreased CT scanning rates through individual provider feedback [24]. They had assembled a team, composed of a nurse educator and research expert, a QI expert, and an administrator, to review the literature on implementation, increase awareness about the PECARN rules and develop strategies to encourage their adoption. A head trauma electronic order set that included a link to the rules and supporting text was also created for support. In our setting, we did not have this support which would improve awareness and adherence to guideline recommendations. In fact, we did not have enough staff (administrative or medical) to implement a true QI project which would include Plan-Do-Study-Act (PDSA) cycles and a multidisciplinary team available to track results and provide feedback. We also did not have Information Technology (IT) support to develop an electronic tool to ease the use of the PECARN rules as previously done [23–25] nor to help generate regular reports that would be used for PDSA cycles and feedback to physicians. Knowing that there is substantial variability in adherence to PECARN rules between physicians worldwide [26]; some of the physicians working in the pediatric ED section in our institution may have been reluctant to adhere to the rules as their adoption is usually influenced by local practice and culture [36]. In addition, the pediatric patients included in this study were evaluated by physicians with a surgical, emergency, or family medicine, rather than pediatric or pediatric emergency, background which have been reported to have higher CT imaging rates [25]. As such, individual provider related factors and limited administrative resources might have weakened the impact of PECARN rules implementation on CT scanning rates in our institution.

Moreover, similar to a nonrandomized multicenter trial [25], the decrease in CT rates in this study was particularly significant among low risk children less than 2 years of age decreasing from 20.7 to 11.4% (*p* = 0.02). Our results are consistent with previous reported findings of an overall higher rate of correctly indicated head CT scans ordered on children less than 2 years of age after implementation of PECARN rules [20, 26]. These findings are noteworthy as children younger than 2 years are the most sensitive to radiation [4]. Specifically, children younger than 2 years with none of the predictor variables for ciTBI have less than 0.02% risk of ciTBI, implying that CT scans are not indicated for most children in this low-risk groups [4]. In our institution, however, before implementation, a substantial proportion (20.7%) of low risk children younger than 2 years were still scanned. Physicians’ certainty in evaluating very young patients is usually lower than for older patients due to the concern of being unable to reliably identify ciTBI. Indeed, the clinical assessment of children less than two years of age is challenging as their neurologic examination is difficult to obtain and interpret; they may be asymptomatic despite having a ciTBI, are at risk for abusive head trauma, and are more prone to skull fractures than older children. Additionally, despite being informed of the clinical inappropriateness and radiation risks of CT imaging, parents often prefer to be reassured with negative results for younger children [37]. As this study’s results show, PECARN rules reduced uncertainty and improved accuracy in medical decision-making and thus provide support for ED physicians to predict which children can be safely managed without CT scanning [38].

All things considered, according to this study with a relatively large sample size in a limited resource setting, despite not having a QI team in place and an ability to monitor things closely, the PECARN rules seem to meet the objective of limiting the use of CT, yet this reduction could be greater by implementing more changes provided additional resources and administrative support are available.

Because structural support has been shown to be effective at supporting reliable change [39], it may be beneficial to create a head trauma electronic order set to remind clinicians of the ciTBI predictors in children with minor head trauma. Future interventions may also include individual provider feedback on CT scanning rates [40, 41] and surveys for PED physicians about causes for failing to adhere to guidelines [42]. Prospective well-designed studies with detailed impact analysis would further support the use of PECARN rules in daily clinical practice. It would then be ideal to implement these changes at a national level, especially given the prevalence of pediatric head trauma.

### Limitations

This is a retrospective single center study, where missing or inaccurate data especially in this history and physical exam findings may not have been accounted for. Moreover, we had no access to the medical records of 37 patients among those who were screened for inclusion, which amount to 2% of the excluded patients. Although we kept the data collection simple, following the PECARN predictors, the reliance on previously documented data might have led to misclassification of patients. Additionally, bounce backs may have presented to outside facilities and may have been missed. However, given that our hospital is the major referral center in our country, bounce backs to other centers would be minimal. Moreover, no standard QI techniques were used to study the effects of implementation. As such, the decrease being a result of the implementation alone is uncertain, however, no other interventions related to care of minor head trauma were implemented at that time.

## Conclusions

PECARN minor head trauma rules’ implementation did not significantly change the overall CT scan rate but reduced the CT scan rate in patients aged < 2 years at low risk of ciTBI. The intervention did not increase the number of missed ciTBI. As such, it is recommended that the PECARN head CT rules be implemented, even if in a simple fashion, in a limited resource setting, as a guide for ED physicians in their clinical decision-making regarding imaging of children with minor head trauma.

## Data Availability

The datasets used and/or analyzed during the current study are available from the corresponding author on reasonable request.

## Abbreviations

AUBMC: American University of Beirut Medical Center
ciTBI: Clinically important traumatic brain injury
CT: Computed tomography
ED: Emergency Department
ICU: Intensive Care Unit
PECARN: Pediatric Emergency Care Applied Research Network
PED: Pediatric Emergency Department
GCS: Glasgow Coma Scale
TBI: Traumatic brain injury
SPSS: Statistical Package for Social Sciences
QI: Quality Improvement
